# The Multi-Functional Roles of CCR7 in Human Immunology and as a Promising Therapeutic Target for Cancer Therapeutics

**DOI:** 10.3389/fmolb.2022.834149

**Published:** 2022-07-06

**Authors:** Faris Alrumaihi

**Affiliations:** Department of Medical Laboratories, College of Applied Medical Sciences, Qassim University, Buraydah, Saudi Arabia

**Keywords:** CC-chemokine receptor 7, CCL19, CCL21, immune tolerance, drug target, prognostic marker

## Abstract

An important hallmark of the human immune system is to provide adaptive immunity against pathogens but tolerance toward self-antigens. The CC-chemokine receptor 7 (CCR7) provides a significant contribution in guiding cells to and within lymphoid organs and is important for acquiring immunity and tolerance. The CCR7 holds great importance in establishing thymic architecture and function and naïve and regulatory T-cell homing in the lymph nodes. Similarly, the receptor is a key regulator in cancer cell migration and the movement of dendritic cells. This makes the CCR7 an important receptor as a drug and prognostic marker. In this review, we discussed several biological roles of the CCR7 and its importance as a drug and prognostic marker.

## Background

To ensure efficient functioning of the immune system, the interaction between immune and non-immune cells is imperative (Stockis et al., 2019). These cellular encounters greatly rely on the cells' ability to migrate to a defined site (Förster et al., 2008). The trafficking of immune cells is regulated by key regulators known as chemokines (Hughes and Nibbs, 2018). Some of these chemokines are produced during infection, while others such as CC-chemokine ligand 21 (CCL21) and CCL19 are expressed every time and function to control cell movement (Förster et al., 2008). Both CCL21 and CCL19 act as sole ligands for a CC-chemokine receptor 7 (CCR7) (Hauser and Legler, 2016). The CCR7 protein is the product of the CCR7 gene and is recently designated as a cluster of differentiation 197 (CD197) (Cuesta-Mateos et al., 2021). Different cells of the immunity system are responsible for CCR7 expression and along with its ligands play a key part in localizing antigen-presenting dendritic cells and T cell subpopulation to lymph nodes, where the cells establish close contacts to drive activation of antigen presentation (Lipscomb and Masten, 2002). The CCR7 is implicated in optimal induction of protective immunity and also for the stimulation of peripheral tolerance induction and immunity response regulation by CD4^+^CD25^+^ regulatory T cells (Cools et al., 2007) (Kondo et al., 2019).

## CCR7 and Its Binders

There are two ligands for CCR7; CCL19 and CCL21. In order to have avid binding to glycosaminoglycans (Rot, 2010), CCL21 has a unique 12 basic amino acid patch in the long C-terminal tail of 32 residues (Proudfoot et al., 2017). The binding event is a prerequisite for effective presentation of CCL21 on endothelial cell surfaces (Miyasaka and Tanaka, 2004). The CCL21 presentation is specifically carried out by podoplanin, which is a proteoglycan expressed by different cell types and might regulate CCL21 availability (Johnson and Jackson, 2010). In mouse experimentation, due to gene duplication, two functional CCL21 variants have been noticed (Zlotnik et al., 2006). One is CCL21-Leu with leucine at position 65 and is expressed by the colon, lung, stomach, skin, and heart (Schumann, 2011). On the other hand, CCL21-Ser is expressed by lymph nodes, thymus, and spleen (Mori et al., 2001). It is interesting to know that the human genome only encodes CCL21-leu and not CCL21-Ser (Hauser and Legler, 2016). The CCL21 in humans and mice is yielded by fibroblastic reticular cells and endothelial venules (Link et al., 2011) (Al-Jokhadar et al., 2017) (Seth et al., 2011). The CCR7 is made of seven transmembrane domain containing proteins and facilitates its signaling pathways through heterotrimeric G proteins (Maghazachi, 2005). The expression of CCR7 is carried out by thymocytes, mature and semi-mature dendritic cells, regulatory T-cells, naïve T-and B-cells, and central memory T-cells (Schneider et al., 2007). In addition, CCR7 expression is carried out by different malignant cells. For CCR7, CCL19 and CCl21 had shown the same binding affinities though they initiate various singling pathways leading to different impacts (Müller et al., 2003). The CCL19 in contrast to CCL21 activates CCR7 internalization and phosphorylation, which shorten the time span of CCR7-mediated cell responses to CCL19 (Hauser and Legler, 2016). Similarly, the CCL19 can desensitize the CCR7 in its subsequent response to CCL21 ligation (Zidar et al., 2009). Together with CCL25, CCL19 and CCL21 have to potential to bind with high affinity to CC-X-chemokine receptors, which act as chemokine interceptors by internalizing ligands and transporting them (Förster et al., 2008).

## Multifunctional Roles of CCR7 in Host Immunology

### Significance of CCR7 in Immune Cell Regulation

**FIGURE 1:**
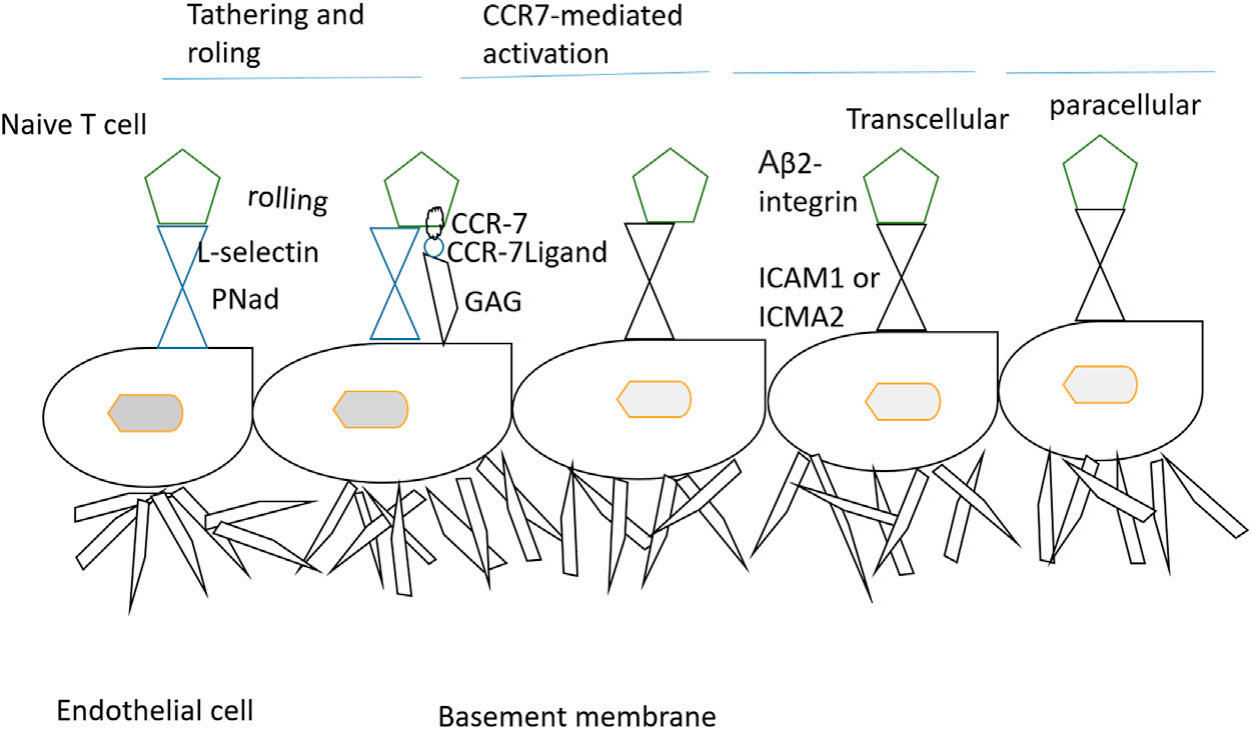
Stepwise mechanism of lymphocyte homing to the lymph nodes. The T-cells when emerging from the blood enter the peripheral lymph nodes through the tethering and rolling mechanism, activation of CCR7, firm arrest, and transendothelial migration. At first, L-selection of lymphocyte binds with peripheral node addressins (PNAd) and sialomucins on high endothelial venules (HEVs). The interaction results in T-cell attachment to HEVs and results in cell rolling. The rolling cells then interact with CCL21/CCL19 and thus are immobilized by glycosaminoglycans (GAGs). The signals from CCR7 and blood flow force induce conformational changes in α_L_β_2-_integrins, thus allowing firm binding to intracellular addressin cell adhesion molecular 1 (ICAM1) and ICAM2. CCR7 also activates α_L_β_2-_integrin mucosal addressin cell-adhesion molecule 1 (MAdCAM) (Förster et al., 2008).

The localization of immune cells to defined functional compartments is controlled by CCR7-mediated signals (Worbs and Förster, 2007). The majority of the T-cells such as memory, naïve, and regulatory T-cells are allowed to penetrate lymph nodes involving a stepwise procedure of interaction of adhesion to endothelial cells (Nolz et al., 2011). In mice experimentation, CCR7 deficiency results in lack of T-cells in lymph nodes (Okada et al., 2002). It was also observed that T-cells are unable to home the lymph nodes but localize to the spleen in the absence of functional CCR7 (Sharma et al., 2015). The B cells in the CCR7-deficient case have the potential to migrate to splenic white pulp and lymph nodes (Katagiri et al., 2004). Though the CCR7 as a receptor of lymph node homing is well-established, evidence suggesting its role in lymphocyte recirculation is also very real (Link et al., 2007). The emigration of T-cells to peripheral tissues and entrance of T-cells to lymph nodes is also a CCR7-dependent step (Ebert et al., 2005). The dendritic cells are present as sentinels in the skin and alimentary, respiratory, and urogenital tracts (Hendry et al., 2017). The activation of dendritic cells by an infectious agent or inflammatory events drives the cells to undergo maturation, resulting in major changes in antigen uptake and presentation (Stockwin et al., 2000). The maturation of dendritic cells can be categorized by the higher expression of CCR7 and CD80, CH83, and CD86 (Chiesa et al., 2003). Very less is known about the exact mechanism of how trafficking of dendritic cells *via* different lymphatic events occurs (Alvarez et al., 2008). Furthermore, it is still under investigation how CCR7 and its ligands mobilize the dendritic cells (McKenna et al., 2005). Both the wild and CCR7-deficient mice were reported to have the same dendritic cell numbers in the peripheral organs (del Rio et al., 2007). This implies that CCR7 has no direct involvement in dendritic cell progenitor recruitment to mucosal and skin surfaces (Cutler and Jotwani, 2004). The migration ability of differentiated dendritic cells from bone marrow to lymph nodes is a major hinderance in CCR7-deicient mice (León et al., 2005). It is also analyzed that the turnover of dendritic cells from the lung, skin, and intestine depends on the CCR7 (Hintzen et al., 2006). In *in vivo* studies, it has been demonstrated that CCL19 and CCL21-Ser derived from lymph nodes take part in activating dendritic cell relocation into the lymph nodes (Denton et al., 2014). CCL19 and CCL21 are needed for dendritic cell guiding in the lymph nodes. Furthermore, research findings speculated that CCL19 and CCL21 are capable of priming T cells along with driving the dendritic cell migration. The uptake of antigens by mature dendritic cells is facilitated by CCR7 ligands (Seubert et al., 2008). A graphical illustration of the stepwise process of lymphocyte homing to the lymph nodes is provided in Figure 1.

### The Role of CCR7 in Lymph-Node Homing

Upon entrance into the lymph node, naïve T-cells begin to migrate in a random walk pattern in the paracortical T-cell-rich area (Krummel et al., 2016) (Weninger et al., 2003). The CCR7-deficient T cells in popliteal lymph nodes have shown 30% reduced velocity as well as 50% reduced motility coefficient (Worbs et al., 2007). Furthermore, a notable dichotomy has been observed within the lymph nodes for chemokine receptor usage (Garcia et al., 2005). The CCR7 activates signals that allow the cell to migrate into the T-cell areas (Arnold et al., 2007). Upon activation, the follicular B-cells upregulate CCR7 and downregulate CXXR5. The differential chemokine receptor expression drives the movement of follicular B-cells to the T-cell zone to get help from CD4^+^ T cells (Eisenbarth et al., 2021). The expression of CCR7 on CD4^+^CXCR5^+^ follicular T cells permits the cells to enter into B cell follicles for providing help in antibody production and class switching (Hardtke et al., 2005). Overall, it can be concluded that CCR7 is a lymph-node receptor for dendritic cells and T-cells.

### The Role of CCR7 in Immune Tolerance

The weak immunity in CCR7-deficient mice after administration of a model antigen further illustrates the multifaceted role of CCR7 and its ligand molecules on the immune system and their vital importance in paracortical area organization in the lymph node (Worbs and Förster, 2007). Studies have also shown that the CCR7-deficient mice impaired humoral immune responses in case of low antigen against replicating virus and high amount of virus glycoproteins (Scandella et al., 2007). These findings imply that when the antigen is sparse, CCR7 holds significant importance in interactions among immune cells (Qi et al., 2006). In some cases, the CCR7-mediated interactions are bypassed in providing adaptive immunity against a pathogen (Moretta et al., 2008). This was highlighted in CCR7-deficient mice where neutralizing immune responses were seen mounted against the choriomeningitis virus (Junt et al., 2008). It was also observed that for priming the naïve MHC-class-Ia-restricted CD8^+^ T cells, the presence of CCR7 is required, whereas MHC-class-II-restricted CD4^+^ T cells and naive MHC-class-Ib-restricted CD8^+^ T cells do not require chemokine receptor (Tzelepis et al., 2007). In addition, it was revealed that repeated administration of tetanus toxoid stimulated humoral and full-blown cellular immunity in CCR7-deficient mice (Macpherson et al., 2008). In auto-immune encephalitis, allergic asthma, and inflammatory bowel disease, substantial immune responses in mice were developed in the absence of CCR7 and its ligands (Griffith et al., 2014). The nonstop migration of dendritic cells from the periphery is a critical step in inducing immune tolerance in response to any food or environmental antigen (Zhang et al., 2021). The migration of tolerogenic or semi-mature dendritic cells into draining lymph nodes depends on CCR7 expression (Förster et al., 2012). This was tested in CCR7-deficient mice whether dendritic cell–mediated transportation of harmless antigens is required for peripheral tolerance (Worbs et al., 2006). The use of intravenous or subcutaneous injection of model antigen ovalbumin in wild-type mice results in systematic non-responsiveness toward model antigen ovalbumin (Steenblock et al., 2009). The mesenteric lymph node was identified as a site of antigen presentation to T cells (Buettner and Bode, 2012) (Jang et al., 2006). Further clarity on the point was obtained from studies where antigen delivery to the respiratory tract is carried out by intratracheal instillation or inhalation (Lombry et al., 2004). The antigen was labeled with fluorochrome to monitor its *in vivo* and *ex vivo* experimentations. The CCR7-deficient mice showed no effect of model antigen ovalbumin aerosol on reporter T-cells (Förster et al., 2008). Therefore, it can be summarized that under homeostatic conditions, the dendritic cells at mucosal sites can induce tolerance in the presence of CCR7 by sampling antigens and transporting them to draining lymph nodes to be efficiently presented to T-cells (Seth et al., 2011).

**FIGURE 2:**
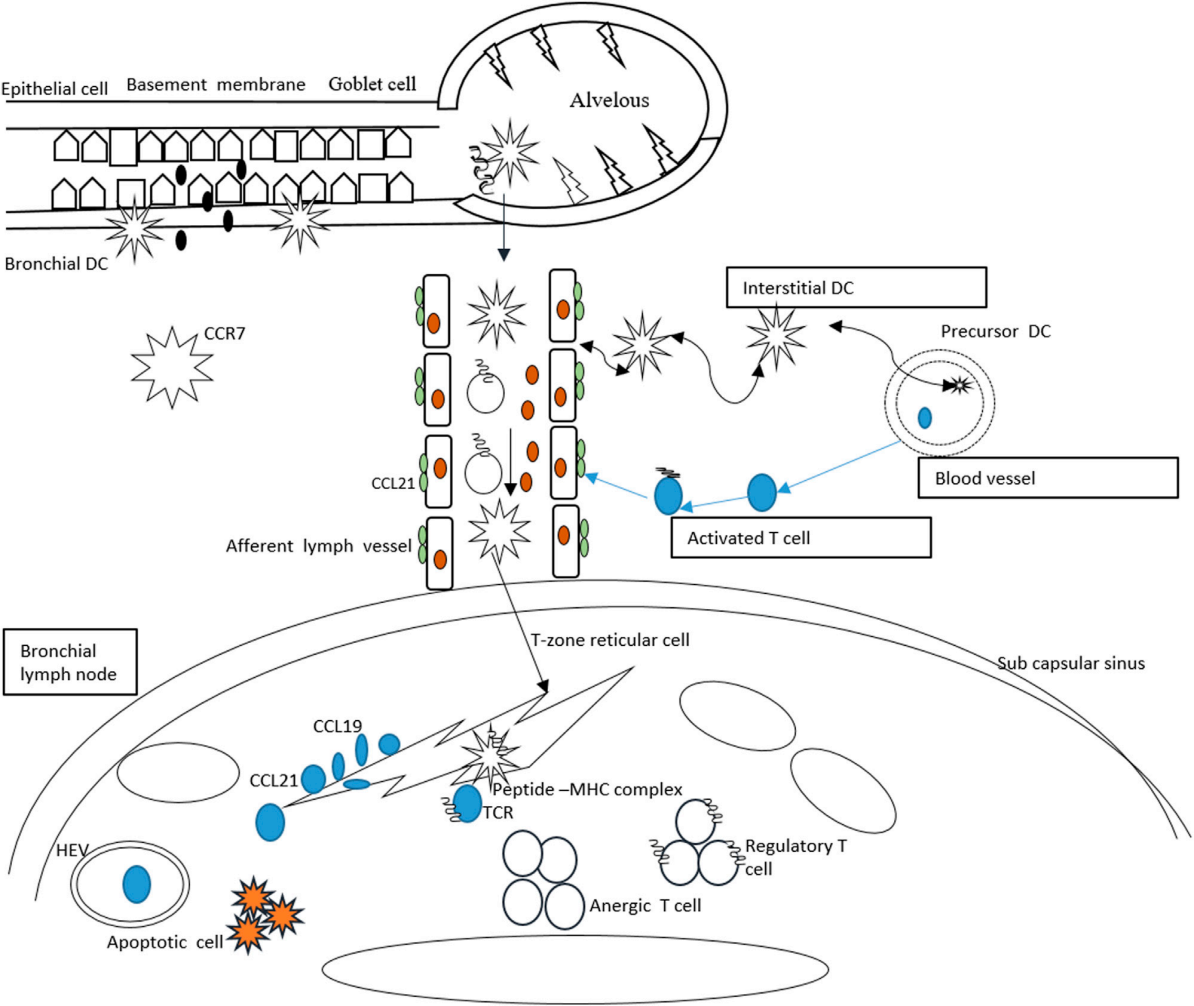
CCR7-mediated tolerance in response to inhaled antigens. The dendritic cells enter the lungs and produce interstitial and bronchial dendritic cells. The bronchial dendritic cells carry the antigens and upregulate the CCR7 and move toward lymphatic vessels to activate CCL21. The dendritic cells are passively transported into the draining lymph node. The dendritic cells then present antigens to the naïve T-cells and *via* endothelial venules, the T-cells penetrate into the lymph node. Migration of T-cells on reticular cells results in expression of CCL19/21.

Suppression of the host immunity through forkhead box P3 (FOXP3) T-cells is considered an alternative method for efficient peripheral immune tolerance to foreign and self-antigens (Nishikawa and Sakaguchi, 2010). The regulatory T-cells can be naturally produced in the thymus when CCR7 is absent. In both wild and CCR7-deficient mice, the total number of FOXP3^+^ T-cells is the same (Schneider et al., 2007) (Smigiel et al., 2014). This can be rational that *in vivo* the cells are unable to reach the lymph nodes and incapable of placing themselves in the T-cell zone (Groom et al., 2012). In the lymph nodes, the exact mechanism behind the regulatory T-cell suppressive activity is still unknown (Wei et al., 2018). The regulatory T-cell homing T-cell zone of the lymph node is mediated by CCR7, proliferates, and expands when they encounter their cognate antigen (Schneider et al., 2007). Reduced number of activated T helper cells due to CCR7-dependent presence of regulatory T-cells is observed (Bayry et al., 2007). Schematically, the CCR7-mediated immune tolerance is presented in Figure 2.

### The Role of CCR7 in Autoimmunity and Lymphoid Neogenesis

**FIGURE 3:**
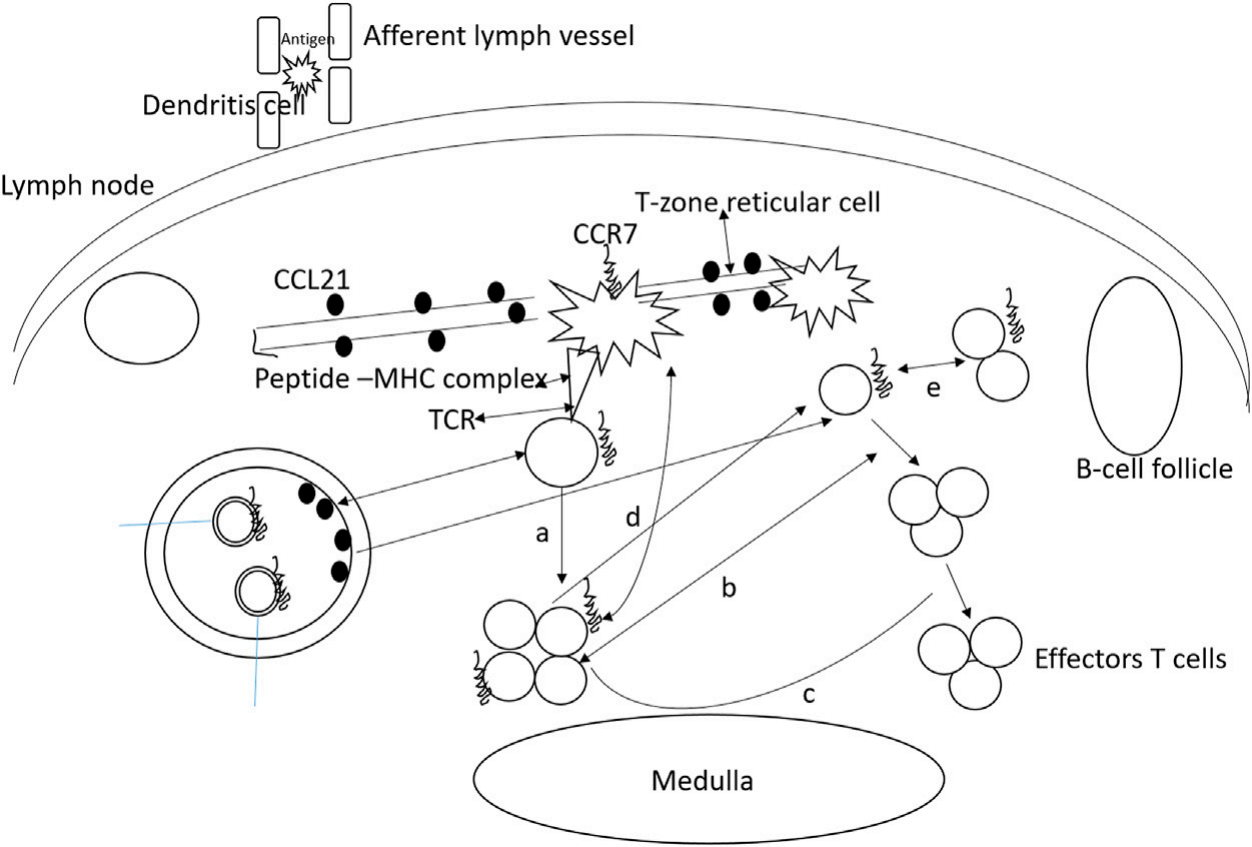
Functioning of CCR7 in regulatory T-cells. Almost all regulatory T-cells express CCR7 and use it for entering into lymph nodes. These cells homing to the lymph node allow their interaction with the antigens on the dendritic cells. As a result, the regulatory T-cells proliferate and expand upon presentation with the antigen. **(A)** Interfere with naïve T helper cell proliferation, **(B)** low number effector cells and inhibits differentiation, **(C)** regulatory T-cells exert suppressive activities by targeting dendritic cells, and **(D)** T-cells. **(E)** naive T cell conversion to regulatory T cells in the presence of low amount of antigens.

It has been observed that the absence of CCR7 is directly associated with the onset of spontaneous autoimmunity. This was evaluated in CCR7-deficient mice where lymphocyte infiltration was reported in different peripheral organs along with high auto-antibody titer resulting in IgG deposition in renal glomeruli. Further investigation reported that the emergence of autoimmunity is the product of ineffective negative selection of autoreactive T cells, defective regulatory T cell function, and lack of proper peripheral tolerance maintenance. It was also noticed that CCR7-deficient mice develop lymphoid at sites such as the stomach, lung, and colon; however, it is not exactly known about the extent of ectopic lymphoid structure contribution to autoimmunity establishment and maintenance. In the absence of CCR7, spontaneous lymphoid neogenesis is also witnessed emphasizing the fact that CCR7 is not needed for the process. Tertiary lymphoid structures are also formed due to transgenic expression of CCR7 in the pancreas and thyroid. Furthermore, tertiary lymphoid structure development in different organs is correlated with CCL21 ectopic expression in infection and autoimmunity. This process is hypothesized to be mediated by CCR7 as tertiary lymphoid structures are not formed in CCR7-deficient mice expressing CCL21. The functioning of CCR7 in regulatory T-cells is presented in Figure 3.

### The Role of CCR7 in Thymus

**FIGURE 4:**
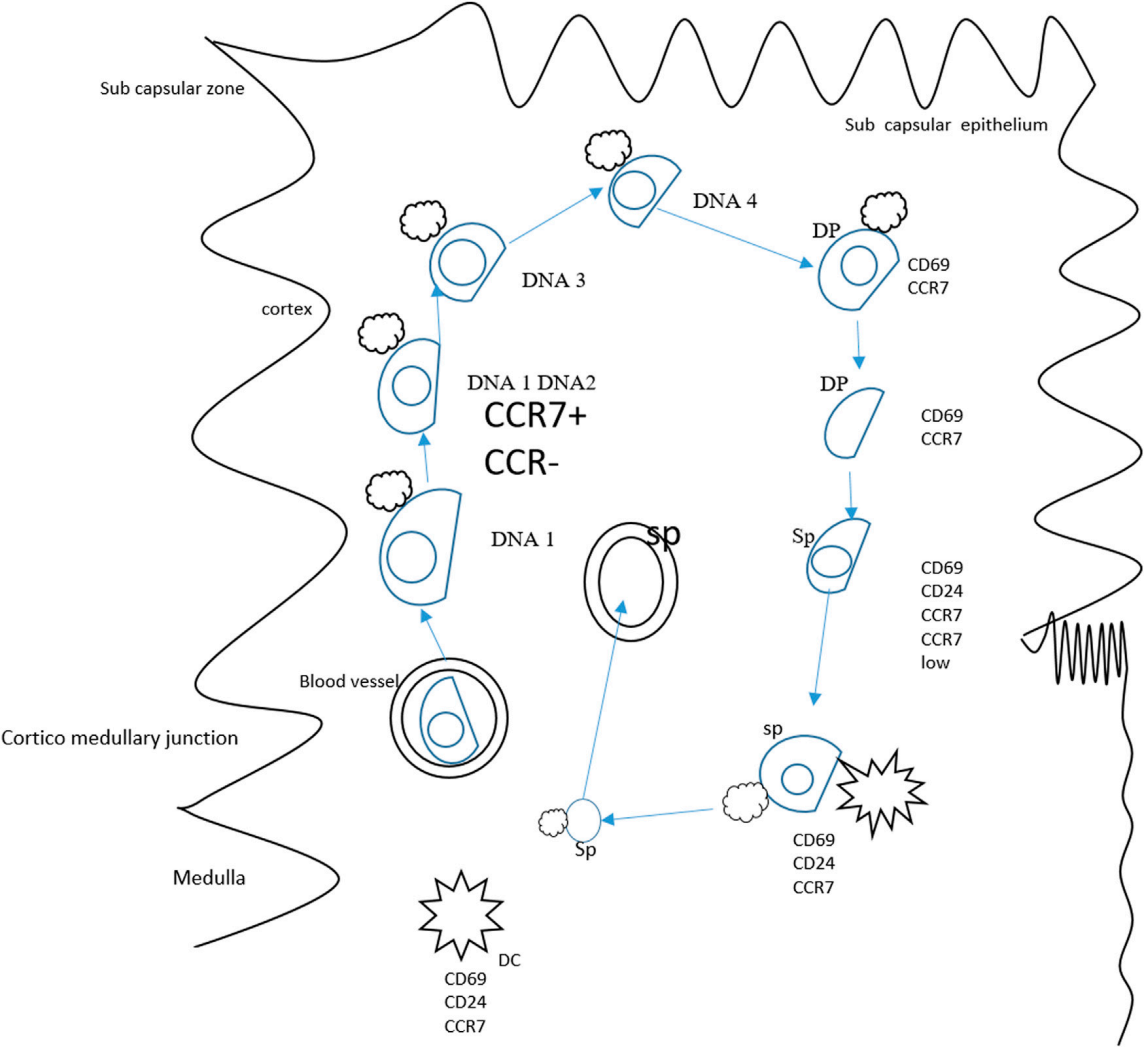
Role of CCR7 in the migration of thymocytes. Thymocyte progenitors derived from bone marrow travel to the thymus. As the CD4/8 lacks expression, the cells are known as double-negative cells. The DN1 cells differentiate at the thymic entry site, and transformation to DN2 occurs in the mid cortex. The DN3 thymocyte differentiation happens while cells migrate to the outer cortex and developed into DN4 cells in the sub-capsular zone. The double-negative dendritic cell transition to the double-positive phase is accomplished in reverse migration and the double-positive thymocytes enter the medulla. In the medulla, positively double-positive cells mature and result in the production of CD4^+^/CD8^+^. A small population of double-positive cells expresses CCR7 and might drive the migration of double-positive cells to the medulla from the cortex. The CCR7 also plays a key role in mature single positive CD62L cells and guides the maturation of these cells. In this period, thymocytes interact with dendritic cells and medullary and thymic epithelial cells, deleting auto-reactive thymocytes and guiding positive selection.

The thymus is an important organ that maintains the pool of peripheral T cells. The CCR7 is revealed to be vital for organizing migratory events of cells in the thymus (Bunting et al., 2011). During embryogenesis, the CCL21 is reported to be involved in fetal hematopoietic progenitor recruitment in developing organs (Liu et al., 2005). The statement can be supported by the fact that CCR7-deficient mice are found to have a reduced number of thymocytes (Laan et al., 2009). Studies have also revealed that mouse overexpression of CCX-CKR possesses a low number of hematopoietic precursors in the thymic region (Bunting et al., 2013). The CCL19 and CCL21 in the adult thymus are not restricted to any compartment and are detectable in the medulla and cortex (Kwan and Killeen, 2004). As a result, CCR7 ligands are capable of guiding developed thymocyte migration through thymic compartments (Kwan and Killeen, 2004). The CD4 and CD8 expression in early progenitors is absent, and the cells are referred to as double negative cells (Ceredig and Rolink, 2002). The expression of CCR7 is prominent in the double-negative subpopulation cells (CD44^hi^ CD25^int^) (Bulati et al., 2014). About fifty percent of these cells express CCR7 reflecting the role of CCR7 in cell migration from cortico–medullary junction (Braun et al., 2011). Recently, the CCR7 role in the translocation of double-positive thymocytes has been studied (Kwan and Killeen, 2004). The CCR7 expression is abundant in single-positive populations (Castro et al., 2014). These cells are found in high concentrations in the medulla. Interestingly, the immature CD4^+^ single-positive cells express very low CCR7 (Kurobe et al., 2006). On the other hand, immune cells that do not undergo negative selection and are mature produce a high amount of CCR7 (McDonald et al., 2015). Another important role of CCR7 expression is the mature thymocyte positioning near blood vessels prior to leaving the thymus (Kwan and Killeen, 2004). The thymus morphology disruption is the result of central tolerance breakdown and autoimmunity development (Lomada et al., 2007). During T cell production in the thymus, the absence of CCR7 signaling contributes to autoimmunity manifestation in CCR7-deficient mice. Along with this, it is also elucidated that CCR7-deficient mice reported defects in negative selection, which might be due to impaired T cell receptor stimulation, and this further signifies the contribution of CCR7 in central tolerance maintenance (Davalos-Misslitz et al., 2007). The role of CCR7 in the migration of thymocyte is given in Figure 4.

## Role of CCR7 in Tumor Growth and Expansion

CCL19 and CCL21 are mostly expressed during the growth of lymphatic vessels and also in other lymphatic organs (Krishnamurty and Turley, 2020) (Wirsing et al., 2018). Disparate CCL19 and CCL21 bind to glycosaminoglycans (GAGs) and immobilize on endothelial cells (Jørgensen et al., 2021). Remarkably, literature reported that CCR7 stimulation with both CCL21 and CCL19 ligands enhances G-protein activation, migration of cells, signaling pathway of the ERK 1/2, and mobilization of calcium (Rizeq and Malki, 2020). Desensitization of the CCR7 and its activation of ERK are mainly facilitated by β-arrestin, suggesting that the effects of CCL19 may be more transitory than with CCL21 cytokines (van Gastel et al., 2018).

Moreover, semi-mature, CXCR4/CXCL12 expression is directly associated with the directing of cancer cells to the lungs, liver, and lymphatic nodes (Liu et al., 2020). The high-level expression of the CCR7/CCL21 axis has mostly related to metastasis lymph nodes regions, while it also plays a vital role in the progression of several different types of other malignancies, such as breast (Cabioglu et al., 2005), gastro (Mashino et al., 2002), melanoma (Takeuchi et al., 2004), neck (Tsuzuki et al., 2006), lung (Takanami, 2003), hepatocyte (Yang et al., 2018), cervical (Wang et al., 2021), thyroid (Wagner et al., 2008), tonsillar (Takeuchi et al., 2004), colon (Li et al., 2011), and prostate cancers (Berndt et al., 2013) as tabulated in Table 1. In many reported cases of these types of malignant cancers, increased size of tumor and invasions were due to CCR7 (Kodama et al., 2007).

**TABLE 1:** Involvement of CCR7 in different human cancers.

Type	Role	References
Bladder cancer	Invasion, migration, proliferation, and poor prognosis	(Mo et al., 2015; Xiong et al., 2017)
Breast cancer	Lymphogenesis, metastasis, and actin polymerization	(Li et al., 2017; Xu et al., 2017; Rizeq and Malki, 2020)
Colorectal cancer	Metastasis and poor prognosis	(Gao et al., 2019; Nagasawa et al., 2021)
Cervical cancer	Metastasis and poor prognosis	(Dai et al., 2017; Tian et al., 2021)
Gastric cancer	Metastasis and poor survival	(Du et al., 2017; Mao et al., 2017)
Lymphomas	Tumor dissemination and poor prognosis	(Li et al., 2018; Du et al., 2019)
Lung cancer	Metastasis and tumor dissemination	(Kwiecień et al., 2019; Salem et al., 2021)
Head and neck cell carcinoma	Metastasis	(Al-Jokhadar et al., 2017; Liu et al., 2018)
Prostate cancer	Metastasis, tumor growth, and lymphatic metastasis	(Makino et al., 2019; Rizeq and Malki, 2020)
Esophageal cancer	Poor prognosis, angiogenesis, and metastasis	(Irino et al., 2014; Yang et al., 2018)
Melanomas	Metastasis and poor outcome	(Takeuchi et al., 2004; Legler et al., 2014)

## The Role of CCR7 in Cancer Cell Migration

Cellular migration *in situ* and *ex situ* is dependent on the biochemical and physical properties of cells. For cells to come out from the blood veins and adhere to the endothelial layer, the chemokines must need to bind with GAGs located in the extracellular matrix (ECM) (Eble and Niland, 2009). There is an electrostatic type of interaction somewhere in the C-terminal region of the chemokine and is positively charged because of lysine and arginine, whereas GAGs possess a negative charge because of the presence of sulfate and carboxylate residues (Severin et al., 2010). Recent research works have reported that body cells can sense the physical and environmental stimuli and respond by altering cellular expression (Kraning-Rush et al., 2012). In addition, chemokines can enhance relocation toward an increasing meditation of a chemo-attractant (Yang et al., 2005). In mature dendritic cells, for example, immobilized CCL21 causes outgrowth of cell and integrin activation, while mobilized CCL19 and CCL21 increase the chemotaxis process (Haessler et al., 2011).

The migration of WBC and CCR7 (+) malignant cells spreading into secondary lymphatic organs is specifically regulated through the interaction of chemokine–chemokine receptors in the environment, and T-cell migration–mediated CCR7-proteins within SLT is very crucial for activation of T-cells in order to generate adaptive immunity (Castriconi et al., 2018). Exploring the migration response of CCR7 proteins coding T-cells within certain types of chemokine environment will facilitate a better understanding of the process of T-cell migration (van der Woude et al., 2017). A function examination of CCR7 in chemotaxis cells may also be helpful in understanding its function in cancer spreading (Wu et al., 2009).

CCR7 is of particular attention in understanding metastasis due to CD4 positive T-cells and dendritic cells needing expression of CCR7 to migrate with the lymphatic tissue (Roberts et al., 2016). The function of lymphatic organs as the extracellular fluids flow sink; it has been assumed that interstitial fluids flow and CCL21 role in conjunction to monitor the migrating of the cancer cells to lymphatic vessels in the development of metastases of cancer (Angeli and Randolph, 2006).

Several studies have revealed that CCL19 and CCL21 can vigorously drive the chemotaxis migration of CCR7-expressing tumor cells (Kabelitz and Wesch, 2003). Furthermore, CCL21 has also been observed to provoke the production of new lymphoid-like structures (Pitzalis et al., 2014). But, the function of CCL21 throughout tumor progression time remains slightly debatable. CCL21 is one of the effective chemo-attractant for tumor-penetrating white blood cells (Rizeq and Malki, 2020). The latest clinical research study described an increased outcome related to increased infiltration of CCR7 (+) T lymphocytes in advanced colon cell carcinoma (Banerje e et al., 2021). In stomach cancer expression of CCR7, early tumor cells were investigated as the most significant component in the determination of lymph node metastasis in cancers (Nagasawa et al., 2021).

## CCR7 and Angiogenesis

CCR7 has also been linked to the formation of a new lymphoid vessel in breast carcinoma patient samples, but the actual mechanism is still unknown (Leong et al., 2021). This lymphoid angiogenesis is mainly facilitated by VEGF-C and the receptor of VEGFR-3 (Angeli and Randolph, 2006). Certainly, the high-level expression of this growth factor is well-reported in increased lymphoid node metastasis type of cancer (Ray and Cleary, 2017). Remarkably, there are several other types of reported studies signifying that each time cancer cells express CCL21 and increase the level of white blood cell recruitment in a specific subpopulation of T–cells CD8 positive and dendritic cells (Rizeq and Malki, 2020).

## CCR7 as a Potential Drug Target

The transmembrane protein CCR7 is correlated with the spread of cancer to the lymph nodes in colon cancer and thus considered a beneficial therapeutic target (Salem et al., 2021). The structure of CCR7 attached to allosteric antagonist Cmp2105 was explained by Jaeger, Bruenle, and their colleagues (Jaeger et al., 2019). The CCR7 was fused with the 52.8 kDa; protein sialidase NanA to ensure its crystallization, and the crystals were distributed to a resolution of 2.1 Å. Cmp2105 was added to the CCR7 which made it more stabilized, and the IC_50_ of Cmp2105 in membrane-based competition assays was measured by radiolabeled CCL19, and its measured value was 35 NM. Surprisingly, the structure showed that Cmp2105 was found inside an intracellular space at the ends of transmembrane (TM) helices. As compared to CX3CL1 and CCR2, Cmp2105 stabilizes an inactive confirmation of CCR7. The similarity search of the 3-D model of 2.3 million compounds using Cmp2105 resulted in the finding that there were 293 compounds with similar pharmacophores to the Cmp2105. The thermal stability assays identified the top two best matches. One of these two was navarixin, which is also called SCH-527123, and MK-7123 antagonist, which shows larger efficacy and solubility. As navarixin has noticeable antimetastatic activity in colon cancer, soCZzmer, and some other cancers, therefore it is now in phase II clinical trials. Because of this observed antagonistic activity, there is the possibility of navarixin being utilized for preventing metastasis, which may likely contribute to the CCR7 antagonism mechanism. Furthermore, this study of CCR7 attached to an antagonist may provide a good platform for additional investigation of some available CCR7 antagonists.

## CCR7 as a Prognostic Marker

Different research reported CCR7 as a cancer marker, but its effects on the OS of cancer patients are still unknown because different studies have shown distinguished results even in the same type of tumor in different patients, for example, rectal cancer and lung cancer. It is also reported that CCR7 has no notable effects on OS in other tumor types such as gastric cancer and breast cancer and SCCHN (Salem et al., 2021). This study found that the association between CCR7 and the diagnosis of several tumors has not been explained and reviewed yet. So, they conducted a meta-analysis to issue valid medical resources on the diagnostic value of CCR7. This meta-analysis included 30 studies in which there were 3,413 patients having 15 different types of tumors. The conducted meta-analysis showed that higher expression of CCR7 can independently be used as an indicator of poorer OS in patients having a tumor. Increased level of CCR7 was also correlated with the worst PFS; but there was no evidence to detect the association of CCR7 with DFS, RFS, and DSS. To investigate the prognostic value of CCR7 in other tumors, further investigation of the subgroup for overall survival (OS) values was performed and because of limited available data, the subgroup analysis for other values was not performed. The results showed that upregulation of CCR7 magnificently lowered the OS of esophageal and gastric tumors patients. Furthermore, the overexpression of CCR7 indicated poor OS in patients having breast cancer, but this prediction was not significant. Over CCR7 expression in patients with lung cancer predicted an association with the best diagnosis. The numbers of samples were not sufficient, which makes the results insignificant and that was of course one of the limitations of the study. Another factor was the negative prognostic factor in patients having tumors in the urogenital system and digestive system. Due to the limited sample size, the association between expressing CCR7 and tumor prognosis is considered not convincing, which can be improved by enlarging the sample size and some further analysis of the association of CCR7 with the clinical prognostic values.

Apart from CCR7 as a prognostic marker in cancer, there were some shortcomings of the work. First, the number of samples was not sufficient; second, CCR7 expression cutoff values were not the same in all studies, which can decrease the efficacy of the results; and third, the HR values were obtained from survival curves which can produce a statistical error. Significant heterogeneity was shown in this meta-analysis and that could be considered in different important factors, for example, type of tumor, method of analysis, the source of the sample, and cutoff value.

The results of this meta-analysis suggested that in some types of tumors, the overexpression of CCR7 is correlated to the worst prognosis of tumor patients (Zu et al., 2019). Though the predictions show that in lung cancer and colon cancer, the CCR7 expression is related to prognosis, but these results need to be improved (Günther et al., 2005). It is concluded that CCR7 is a good indicator in tumors, and these results should be considered carefully.

## Conclusion

The CCR7 and its ligands have received great attention in recent times due to their versatile functioning in regulating leukocyte function during immunological responses. The chemokine ability to convey signals that are remarkably versatile and specific makes them powerful modulators of immunological responses against diverse antigens. Considering the importance of CCR7, in this review, we seek to address the importance of CCR7 in immune cell regulation, lymph node homing, immune tolerance, different types of cancer, and CCR7 as a therapeutic and prognostic marker. The literature reported herein might attract the readers for expanding their knowledge of chemokines and a better approach to novel therapeutics in the near future.
